# Supplementary material for: Comparative multiomics analysis of cell physiological state after culture in a basket bioreactor
Source: Sci Rep. 2022 Nov 23;12:20161. doi: 10.1038/s41598-022-24687-4 (PMC9686226; doi:10.1038/s41598-022-24687-4)
Supplement: Supplementary file 1 — Supplementary Information 1. [file 41598_2022_24687_MOESM1_ESM.zip › raw data/Proteomic raw data/6.KEGGenrichment/EG.vs.CG/DOWN/pathway/src/04810.html]

map04810


- K05737

- Down regulated proteins

A0A0D9R3N7(Description:GIT ArfGAP 1;Gene:GIT1;log2FC:-1.2862)

- K05718, K05719, K06461, K06462, K06464, K06476, K06480, K06481, K06482, K06483, K06484, K06485, K06487, K06493, K06524, K06525, K06583, K06584, K06585, K06586, K06587, K06588, K06589, K06590, K06591, K06594

- Down regulated proteins


A0A0D9QZZ6(Description:VWFA domain-containing protein;Gene:ITGAX;log2FC:-1.9952)

- K05717

- Down regulated proteins

A0A0D9R9X4(Description:Fibronectin;Gene:FN1;log2FC:-1.1754)

Close
